# ALDH1 inhibition by DIMATE maintains immune responses and promotes immunogenic remodeling in AML

**DOI:** 10.3389/fimmu.2026.1876637

**Published:** 2026-08-12

**Authors:** Céline Baier, Julien Colle, Yasmine Labiad, Pascal Rihet, Mileidys Pérez-Alea, Béatrice Loriod, Ismail Ceylan, Geoffroy Venton, Guillaume Martin, Régis Costello

**Affiliations:** 1 Aix Marseille Univ, Theories and Approaches of Genomic Complexity (TAGC), INSERM UMR1090, Parc Scientifique de Luminy, Marseille, France; 2 Advanced BioDesign, Parc Technologique de Lyon, Saint Priest, France; 3 Service Hématologie & Thérapie Cellulaire, Assistance Publique-Hôpitaux de Marseille (AP-HM), Hôpital de la Conception, Marseille, France; 4 Aix Marseille Univ, Transcriptomique et Génomique de Marseille-Luminy (TGML) Platform, Parc Scientifique de Luminy, Marseille, France

**Keywords:** ABD-3001, acute myeloid leukemia (AML), ALDH1 inhibitor, DIMATE, immunogenic cell death (ICD), tumor immunogenicity

## Abstract

Acute myeloid leukemia (AML) treatments often cause profound immunosuppression, limiting immune-mediated control of residual disease. DIMATE, an ALDH1 inhibitor under clinical evaluation, induces aldehyde and redox stress in leukemic cells, but its impact on immune effector functions and tumor immunogenicity remains insufficiently defined. Here, we assessed the effects of DIMATE on human immune cells and AML models, including peripheral blood mononuclear cells, AML cell lines, and primary AML samples. DIMATE largely preserved selected immune effector functions at pharmacologically relevant concentrations, including T-cell activation, natural killer cell cytotoxicity, and phagocyte oxidative burst. In parallel, DIMATE induced pro-inflammatory and endoplasmic reticulum stress pathways in AML cells, upregulated co-stimulatory molecules, and promoted ICD-compatible features, including ecto-calreticulin exposure. These findings suggest that DIMATE may couple immune preservation with enhanced leukemic immunogenicity, supporting its potential for combination with immune-engaging therapies and strategies targeting measurable residual disease in AML, but also as a single agent capable of fostering a more immunostimulatory anti-leukemic context.

## Introduction

Acute myeloid leukemia (AML) treatment has traditionally relied on high-dose chemotherapy or, more recently, on combinations of a hypomethylating agents with the BCL-2 inhibitor venetoclax. Both approaches are associated with profound and prolonged immunosuppression. The resulting high-grade cytopenias increase the risk of life-threatening infections, which can lead to severe sepsis, organ failure, and death (1). These regimens also markedly elevate the incidence of opportunistic infections, sometimes persisting for years after therapy completion. Over the past decade, the limitations of such immunosuppressive approaches have become evident, especially with the emergence of therapies that interact with and harness the host immune system (2, 3). In this context, anticancer agents with minimal immunosuppressive effects would represent a valuable addition to our therapeutic armamentarium.

Deploying a drug that spares immune function could improve the treatment safety for AML patients, potentially reducing infectious complications and treatment interruptions due to severe adverse events. Moreover, immune-preserving strategies might enhance responses to antibody-based therapies that are increasingly explored in AML, such as ICT01, an anti-butyrophilin 3A (BTN3A) monoclonal antibody that selectively activates γ9δ2 T cells (4). Beyond induction therapy, agents that do not impair immune competence could also facilitate measurable residual disease (MRD) control, given the pivotal role of host immunity in long-term disease surveillance, an effect indirectly supported by the graft-versus-leukemia (GVL) activity observed in allogeneic hematopoietic stem cell transplantation (allo-HSCT) (5, 6). Finally, by preserving immune cells from toxicity, such therapies may allow immune-stimulatory signaling to re-activate antitumor immunity, notably through the induction of immunogenic cell death (ICD), a regulated cell death modality that elicits host immune responses against tumor neo-antigens (7).

We previously reported the anti-leukemic activity of DIMATE, an inhibitor of aldehyde dehydrogenase (ALDH), as a novel therapeutic approach (8). DIMATE is currently under clinical investigation in AML in the First-In-Human ODYSSEY trial (NCT05601726). ALDH enzymes catalyze the conversion of all-trans retinal into retinoic acid (RA), which activates nuclear receptors to regulate gene expression (9). The ALDH1 subfamily also detoxifies lipid peroxidation by-products, including acrolein, 4-hydroxy-2-nonenal (4-HNE), and malondialdehyde (10) which is generated during arachidonic acid lipid peroxidation under reactive oxygen species (ROS) stress. In cancer, ROS can be co-opted to promote anti-apoptotic signaling and contribute to immune evasion (11, 12). By inhibiting ALDH1, DIMATE increases intracellular aldehyde accumulation, which in turn triggers a vicious cycle in cancer cells: glutathione depletion, enhanced ROS formation (notably H_2_O_2_, which fuels further lipid peroxidation and aldehyde generation), and disruption of redox homeostasis, ultimately culminating in cell death (13).

Preclinical studies have shown that DIMATE eradicates leukemia stem cells (LSCs) while sparing normal hematopoietic stem cells (NHSCs). In LSCs (CD45^+^CD34^+^CD38^-^ALDH^+^), the IC50 of DIMATE was 2.8 μM (8). In NHSCs, no overt toxicity was observed up to 10 μM, thereby defining a practical concentration window between 2.8 μM and 10 μM. These findings were established both *in vitro* in human normal progenitors and *in vivo* in AML mouse models (8, 13). Nevertheless, despite compelling anti-leukemic effects, the impact of DIMATE on immune cell populations and functions, a key component of the tumor microenvironment (TME), remains insufficiently characterized.

Emerging literature suggests that nitro-oxidative stress dynamics are integral to the immune-inflammatory response and may contribute to its efficacy (14). Conversely, T cells possess robust antioxidant systems (e.g., superoxide dismutases, peroxiredoxins, glutaredoxins) that buffer ROS (15), suggesting that ALDH inhibition may not intrinsically suppress their functions. Additionally, ALDH1A-mediated RA production promotes regulatory T-cells (Tregs) (16), and inhibition of the RA pathway has been associated with reduced tumor-infiltrating Tregs (16). Finally, ROS modulation through ALDH inhibition and ER stress may contribute to ICD-associated features, providing antigenic and danger signals that could support antitumor immune responses (17).

Here we report a set of translational immune-pharmacology studies demonstrating that DIMATE (i) preserves key functions of PBMCs, T cells, NK cells, and phagocytes, and (ii) induces ICD-compatible programs and ecto-calreticulin (ecto-CRT) exposure in leukemic cells. These findings provide a mechanistic rationale for combining DIMATE with immune-engaging therapies and for exploring its potential in MRD control. Additionally, these features may be related to infusion-related reactions (IRRs) observed in the ODYSSEY trial, highlighting the need for correlative studies to test this mechanistic link.

## Materials and methods

### Cell lines and reagents

Human AML cell lines HL-60 (DSMZ, #ACC3), MOLM-14 (DSMZ, # ACC 777), KG-1 (DSMZ, # ACC 14), KASUMI-3 (DSMZ, #ACC 714) and K562 (DSMZ, #ACC10) were cultured in RPMI 1640 GlutaMax medium supplemented 10% fetal bovine serum, in a humidified CO2 incubator at 37 °C. DIMATE was provided by Advanced BioDesign (Saint-Priest, France). 3,3’-Dioctadecyloxacarbocyanine perchlorate (DIOC; Sigma-Merck, #D4292-20MG) was obtained from Sigma-Aldrich (St-Louis, MO, USA). We also used propidium iodide solution (Sigma-Merck, #P4170-25MG), dihydrorhodamine 123 (DHR) and the PHAGOBURST™ kit (Glycotope Biotechnology, version 05/12).

### Humans blood samples

For experimentations, we used fresh peripheral blood mononuclear cells (PBMCs) from healthy adult’s blood samples provided by the Etablissement Français du Sang (France). Unless otherwise specified, the culture medium for all the PBMC assays contained RPMI 1640 GlutaMAX supplemented with 20% fetal bovine serum and 10U/ml Il-2 IS (Miltenyi Biotec, #130-097-748). For leukemic cells, peripheral blood samples were collected from 3 AML patients (**Supplementary Table 1**). All patient samples were collected in accordance with ethical guidelines (ethical committee “Comité de Protection des Personnes Sud-Ouest et Outre-mer III, France”), complying with the Declaration of Helsinki, and with informed consent from the patients who authorized the use and publication of their data (ClinicalTrials.gov ID NCT06906978).

### Flow cytometry analysis

All flow cytometer experiments were performed using BD Biosciences LSRFortessa X-20 Cell Analyzer. Briefly, for all cell types (T lymphocytes, NK cells or HL-60), pellet cells were washed one time with PBS and centrifugated at 1200rpm 5minutes. Pellets were suspended in 100µl of PBS 1X with 2µl of antibody. Cells were placed at 4 °C, protected from light, for 30 minutes. They were washed with PBS and assessed by flow cytometry.

The following antibodies were used: anti-CD3-APC (Miltenyi Biotec, #130-110-764), anti-CD4-FITC (Miltenyi Biotec, #130-113-229), anti-CD8-PerCP (Miltenyi Biotec, #130-110-682), anti-CD16-APC-Vio770 (Miltenyi Biotec, #130-106-707), anti-CD56-PE-Vio770 (Miltenyi Biotec, #130-11-313) and Anti-calreticulin (D3E6) rabbit mAb conjugated to Alexa Fluor^®^ 488 (OZYME, #CST62304). All flow cytometry data were analyzed using Kaluza analysis Software v2.1 (Beckman Coulter Life Sciences, Indianapolis, IN, USA).

### PBMCs proliferation

Concentrations of DIMATE ranging from 1 to 20 µM were tested. PBMCs (n = 6) were incubated for 48 hours at a density of 2 × 10^6^ cells/mL in 96-well plates, in 37 °C incubator. Cell viability was assessed using the AlamarBlue cell viability assay. Drug sensitivity was quantified by comparing the proliferation ratio to the T0 signal and is reported as the fold increase relative to untreated cells.

### Leukocyte oxidative burst

100µl of fresh blood samples (n = 6) were first untreated or pre-treated with 5 or 10µM of DIMATE solution for 5 hours in 37 °C incubator. Oxidative burst was directly detected with Phagoburst™ kit, according to manufacturer’s instructions. Briefly, opsonized *E. coli* bacteria and the fluorogenic substrate DHR were added to blood samples. During oxidative burst, DHR is converted into rhodamine 123 which fluoresces when excited by a 488nm laser. A total of 10,000 cell events were collected and analyzed using flow cytometry.

### T cells proliferation and activation assay

PBMCs at the final concentration of 1.10^6^ cells/ml were activated using Dynabeads human T-activator CD3/CD28 (Life technologies, #11131D) at a ratio of 1:1 (beads/PBMC). For T-cell proliferation and activation assays (n = 5), DIMATE concentrations of 10 and 15μM were selected to assess whether sustained exposure to pharmacologically active concentrations could affect adaptive immune-cell function under stringent experimental conditions. 48 hours after initiation of treatment, aliquots of cell culture supernatants (n = 4) were collected and evaluated for IL-2 production by human IL-2 uncoated ELISA (Invitrogen, #88-7025) according to manufacturer’s instructions. The remaining cultures were maintained under incubation for subsequent analyses: up to 216 h for flow cytometric characterization of CD4^+^ and CD8^+^ T-cell subsets, and up to 336 h for longitudinal assessment of T-cell proliferation (n = 5).

### NK cell proliferation & activity assay

For NK-cells proliferation, PBMC (n = 3) were cultured in RPMI 1640 GlutaMAX supplemented with 10% fetal bovine, PHA 5 µg/mL, 1000U/ml Il-2 IS (Miltenyi Biotec, #130-097-748) at a concentration of 1.10^6^ cells/ml. NK-cell proliferation (CD56^+^/CD16^+^) was monitored overnight using a lower concentration (5 μM) was included to evaluate potential effects on innate immune-cell function within a pharmacologically relevant range, while 15 μM was retained as a common high-dose condition to enable comparison across immune-cell subsets. Expression of Natural Cytotoxicity Receptor (NCRs) Nkp46, NKp44 and NKp30 were analyzed with or without DIMATE at the same condition.

For NK cell toxicity assay (n = 6) using K562 as target cells, K562 were suspended in RPMI without phenol red at a concentration of 1.10^6^ cells/ml and labelled by adding 5µl of 1mM DIOC per ml of cells. K562 were incubated at 37 °C in 5% CO2 incubator for 10 min, washed two times in RPMI without phenol red. PBMCs were pre-treated with 5µM and 15µM of DIMATE for 6h in 37 °C incubator at a concentration of 2.10^6^cells/ml. PBMC (effector cells) were added to the DIOC-K562 (target cells) at an effector-target ratio (200:1). Cells were centrifuged for 3 minutes at 120g to ensure cell contact and incubated at 37 °C, 5% CO_2_. After 18 hours, 3µl of PI at 1mg/ml was added before acquisition by flow cytometry. A total of 1,000 DIOC labelled elements were acquired.

### Ectopic expression of calreticulin

Ecto-CRT analysis using flow cytometry was performed by immunostaining. HL-60, MOLM-14, KG-1 and KASUMI-3 and leukemic cells from 3 AML patients were treated with DIMATE 5µM during 24h in 6-well plate at a concentration of 3.10^5^cells/well for cell lines and 1.10^6^cells/well for leukemic cells. Cell lines and primary AML cells were collected and incubated with 2μl of anti-calreticulin (D3E6) rabbit mAb conjugated to Alexa Fluor^®^ 488 or Rabbit (DA1E) mAb IgG XP^®^ isotype control-Alexa Fluor^®^ 488 Conjugate (CST62304 and #CST2975, OSYME) in 100µl of PBS for 30 minutes at 4 °C in the darkness. Cells were then washed and resuspended in PBS, 2μl of Propidium Iodide (1mg/ml) was added before flow cytometry analysis. Quantification of the percentage of ecto-CRT positive cells was conducted with Kaluza by gating on the singulet population.

### RNA extraction and quantitative real-time PCR

HL-60 were plated in 6-well plate at a concentration of 5.10^5^cells/ml and treated with DIMATE at 10µM for 6h. HL-60 were pelleted, and total RNA was extracted using RNeasy Mini kit (Qiagen, France) according to manufacturer’s instructions with DNase treatment. RNAs were quantified with NanoDrop 1000 spectrophotometer (Nano Drop Technologies, San Diego, CA, USA). Optical density was measured at 260 and 280 nm and the ratio 260/280 (> 1.8) indicates its purity. Complementary DNA (cDNA) was synthesized from 1µg of total RNA using the SuperScript™ VILO™ cDNA Synthesis Kit (Thermo Fisher Scientific, Cat# 11755250, Abingdon, UK) following the manufacturer’s recommendations. Quantitative real-time PCR was performed using SYBR™ Green Master Mix (Thermo Fisher Scientific, Cat# 4367660) on a QuantStudio™ 6 Flex Real-Time PCR System (Applied Biosystems). Primers used are listed in **Supplementary Table 3**. Gene expression was normalized to the mRNA levels of the housekeeping genes GAPDH and ACTB.

### Pangenomic gene expression assay

One hundred nanogram of total RNA was labelled using one-color microarray-based gene expression analysis: low input quick amp (LIQA) labelling protocol: 0.6 μg of the purified Cy3 labeled cRNA were hybridized for 17h at 65 °C, at 60rpm, using the SurePrint G3 human GE 8x60K V2 chips (Agilent Technologies, Santa Clara, California). Microarrays were composed of 62–928 features. Probes synthesized on chips had a size of 60 nucleotides. Microarrays were washed using gene expression wash buffer kit (Agilent Technologies) and scanned through standard Agilent protocol. Data was processed using Feature Extraction software.

### Differential gene expression analysis

The library AgiND is implemented in R software to analyze and visualize data. AgiND was developed on Bioconductor library model and is used to diagnose data quality and data microarrays normalization. Quantile method was used to normalize data; the objective was to homogenize distribution of microarray intensity. A filter was applied on row data to delete controls, then a second filter was applied to delete genes which were expressed under the background in 100% of samples in each group, to identify the genes differentially expressed between untreated or treated by DIMATE HL-60 cells. A tree class significance analysis of Microarray (SAM) test was performed on the normalized and filtered data. A multiple test correction was performed, a false discovery rate (FDR) of 5% and a fold change of 1,5 were fixed. A supervised hierarchical clustering was applied on adjusted gene expression median adjusted data to group genes and samples, according to their expression using « TMeV» (Tigr MultiExperiment Viewer) MeV: MultiExperiment Viewer; Part of the TM4 Microarray Software Suite. Pearson correlation was used, and clusters were grouped on basis of average linkage method. The differential expressed genes (DEG) list was extracted and saved to further analysis.

### Statistical analysis

Data was analyzed using GraphPad Prism 7 software (GraphPad Software). Normality of continuous endpoints was assessed using the Shapiro–Wilk test on raw values within each comparison group. For two-group contrasts, normally distributed data were analyzed using unpaired two-sided Student’s t-tests. For comparisons involving more than two groups (e.g., NT and multiple DIMATE concentrations), we applied one-way ANOVA followed, when the global F-test was significant, by Dunnett’s multiple comparisons test using the untreated control as the reference. For ecto-CRT analyses, a two-way ANOVA with Sidak’s multiple comparisons test was used to assess the effects of treatment and cell line and their interaction. Unless otherwise specified, results are presented as mean ± SEM with two-sided p-values and α = 0.05. For multi-gene/transcriptomic analyses, we controlled the false discovery rate (FDR) at 5 % using the Benjamini–Hochberg procedure; genes/pathways were considered differentially expressed at FDR ≤ 0.05 with preset fold-change thresholds. All analyses were performed on independent donors/replicates (biological replicates), with the sample size (N) reported per figure panel. Significance was set at p < 0.05 (or FDR ≤ 0.05 for multi-gene analyses).

## Results

### DIMATE preserves PBMC proliferation and innate oxidative function

**Figure 1:**
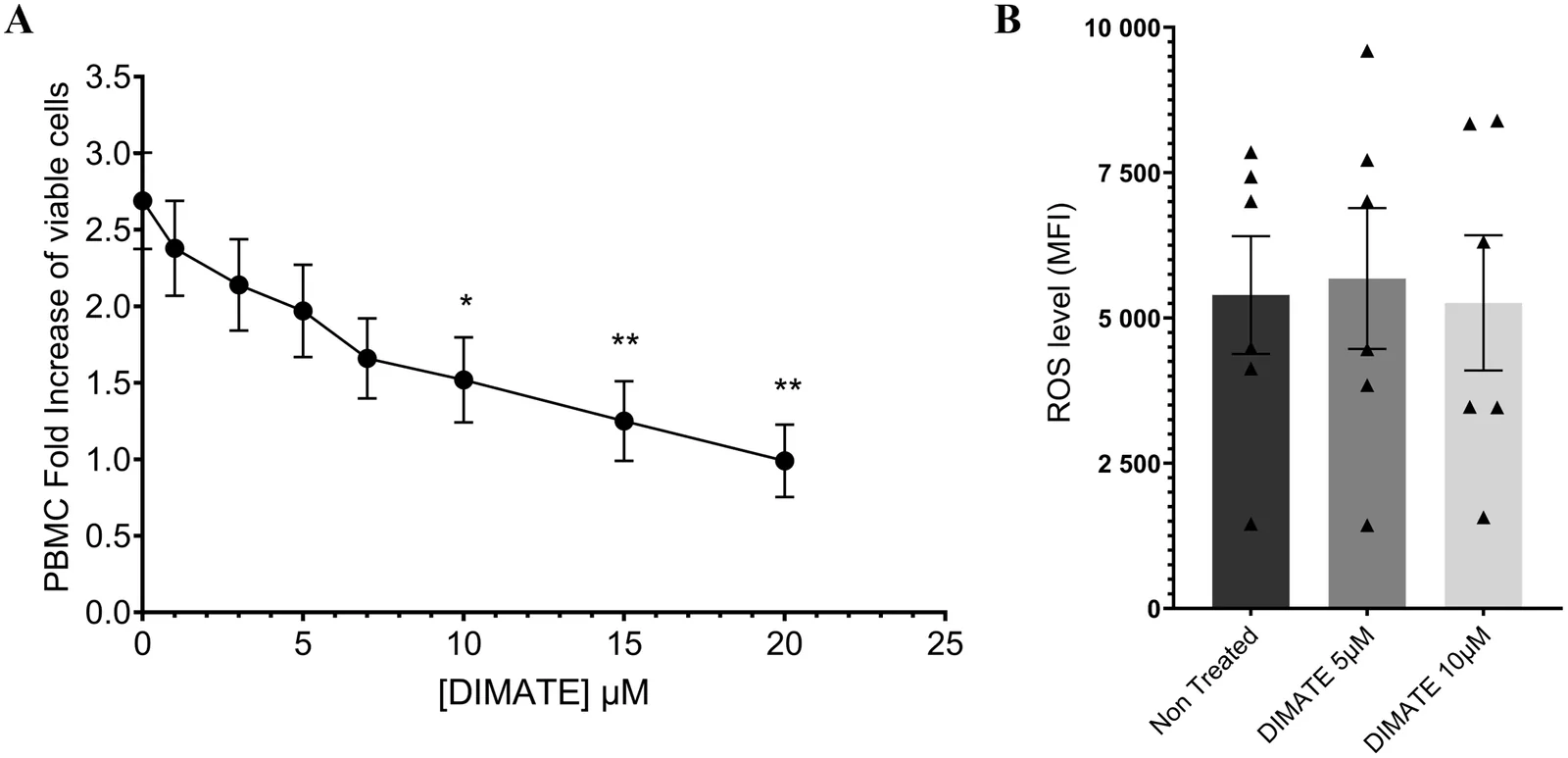
Effect of DIMATE on PBMC proliferation and leukocyte ROS production. **(A)** PBMCs isolated from buffy coats were exposed to DIMATE for 48 h, and the fold-increase (FI) of viable cells was quantified using Alamar Blue (n= 6). A significant reduction in proliferation was observed at ≥ 10µM compared with Non Treated (NT), while no overt cytotoxicity was detected at 20 µM (FI = 0.99 ± 0.58). Data are presented as mean ± SEM. One-way ANOVA with Dunnett’s multiple comparisons versus NT. **(B)** Leukocyte reactive oxygen species (ROS) measured 10 min after stimulation with opsonized bacteria in the presence of DIMATE (5 or 10 µM). Geometric mean MFI: NT 5395 ± 1012 vs 5 µM 5679 ± 1213 (p = 0.86) and 10 µM 5260 ± 1162 (p = 0.93) (n = 6). Data are shown as mean ± SEM. One-way ANOVA with Dunnett’s multiple comparisons versus NT.

We first examined the dose-dependent impact of DIMATE on the proliferation of healthy PBMCs (n=6) (**Figure 1A**). PBMCs were cultured at physiological density (2.0 × 10^6^ cells/mL) and exposed to increasing concentrations of DIMATE following a 48-hour equilibration period in medium alone. Across the 1–10μM range, PBMCs displayed sustained proliferation over 48 hours with no evidence of growth inhibition. At 20 μM, proliferation declined to a mean fold-increase of 0.99 ± 0.58, indicating a cytostatic effect only at this supra-pharmacologic concentration. Collectively, these findings tend to prove that DIMATE preserves PBMC viability and expansion at concentrations ≤10 μM, defining a biologically relevant window for downstream functional assays. Given the central role of monocytes and neutrophils in microbial clearance and antibody-dependent cellular cytotoxicity, we next evaluated whether DIMATE alters ROS production during an *E. coli* challenge (n = 6) (**Figure 1B**). DIMATE at 5 or 10 μM did not impair oxidative burst activity: ROS-associated fluorescence increased from baseline (151.2 ± 38.5 MFI) to 5395 ± 1012 MFI in controls, 5679 ± 1213 MFI at 5 μM (p = 0.86), and 5260 ± 1162 MFI at 10 μM (p = 0.93). These results confirm that innate effector responses, including phagocyte oxidative burst, are not impaired under DIMATE exposure.

### DIMATE spares T-cell effector activation

**Figure 2:**
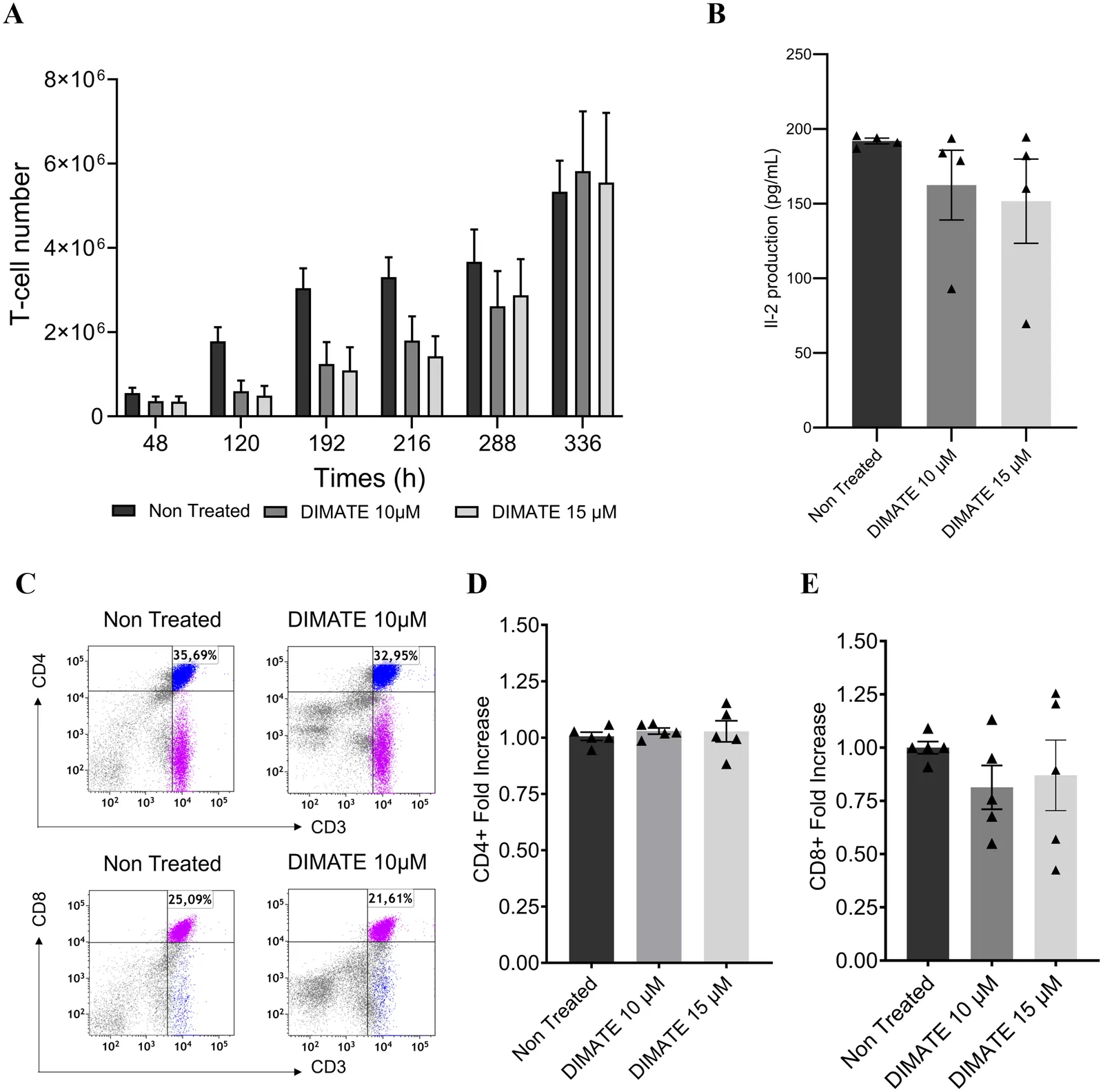
Effect of DIMATE T-cell subpopulations proliferation and activity. **(A)** T-cell expansion from PBMC cultures stimulated with anti-CD3/anti-CD28 was monitored over 360 h with 10 or 15 μM DIMATE; cell numbers were determined by flow cytometry using anti-CD3 labelling (n = 5). Data are shown as mean ± SEM. Repeated-measures two-way ANOVA (factors: time, treatment) with Dunnett’s multiple comparisons vs Non Treated (NT) at each time-point. **(B)** IL-2 production at 48 h post anti-CD3/anti-CD28 stimulation: NT 192.0 ± 3.78 pg/mL vs 10 μM 162.4 ± 46.65 pg/mL (p = 0.535) and 15 μM 151.7 ± 56.48 pg/mL (p = 0.343) (n = 4). Data are presented as mean ± SEM. Statistical significance was assessed using a one-way ANOVA followed by Dunnett’s multiple comparisons test versus the NT. **(C)** Representative T-cell subset distribution (CD3^+^/CD4^+^ in blue; CD3^+^/CD8^+^ in violet) after 216 h with or without 10 μM DIMATE (10 μM vs NT) for CD4^+^ and CD8^+^ frequencies. **(D)** CD4^+^ proliferation at 216 h: NT 1.006 ± 0.041; 10 μM 1.029 ± 0.030 (p = 0.81); 15 μM 1.028 ± 0.105 (p = 0.831) (n = 5). Data are shown as mean ± SEM. One-way ANOVA with Dunnett’s multiple comparisons versus NT. **(E)** CD8^+^ proliferation at 216 h: NT 1.000 ± 0.064; 10 μM 0.814 ± 0.229 (p = 0.429); 15 μM 0.870 ± 0.370 (p = 0.644) (n = 5). Data are shown as mean ± SEM. One-way ANOVA with Dunnett’s multiple comparisons versus NT.

We next assessed whether DIMATE alters T-cell expansion and activation in anti-CD3/CD28-stimulated PBMC cultures (n up to 8) (**Figures 2A–D**). During short-term exposure (0-48 h) to 10–15 μM, T-cell proliferation was preserved. With prolonged incubation (120–288 h), a modest attenuation emerged only at the highest dose, this effect had fully resolved by 336 h (n = 5) (**Figure 2A**). Early IL-2 release (48 h) remained not significantly different from untreated controls (n = 4) (NT 192.0 ± 3.78 pg/mL; 10 μM 162.4 ± 46.65 pg/mL, p = 0.535; 15 μM 151.7 ± 56.48 pg/mL, p = 0.343) (**Figure 2B**). Subset analysis at 216 h showed stable CD4^+^ fractions and a non-significant downward trend in CD8^+^ counts, without changes in CD8^+^ percentages (n =5) (**Figures 2C–E**). Altogether, these findings indicate short-term T-cell activation and expansion were maintained during DIMATE exposure, whereas extended high-dose exposure induced a transient but reversible attenuation without evidence of durable suppression.

### DIMATE does not alter NK-cell proliferation and even promotes cytotoxic activity

**Figure 3:**
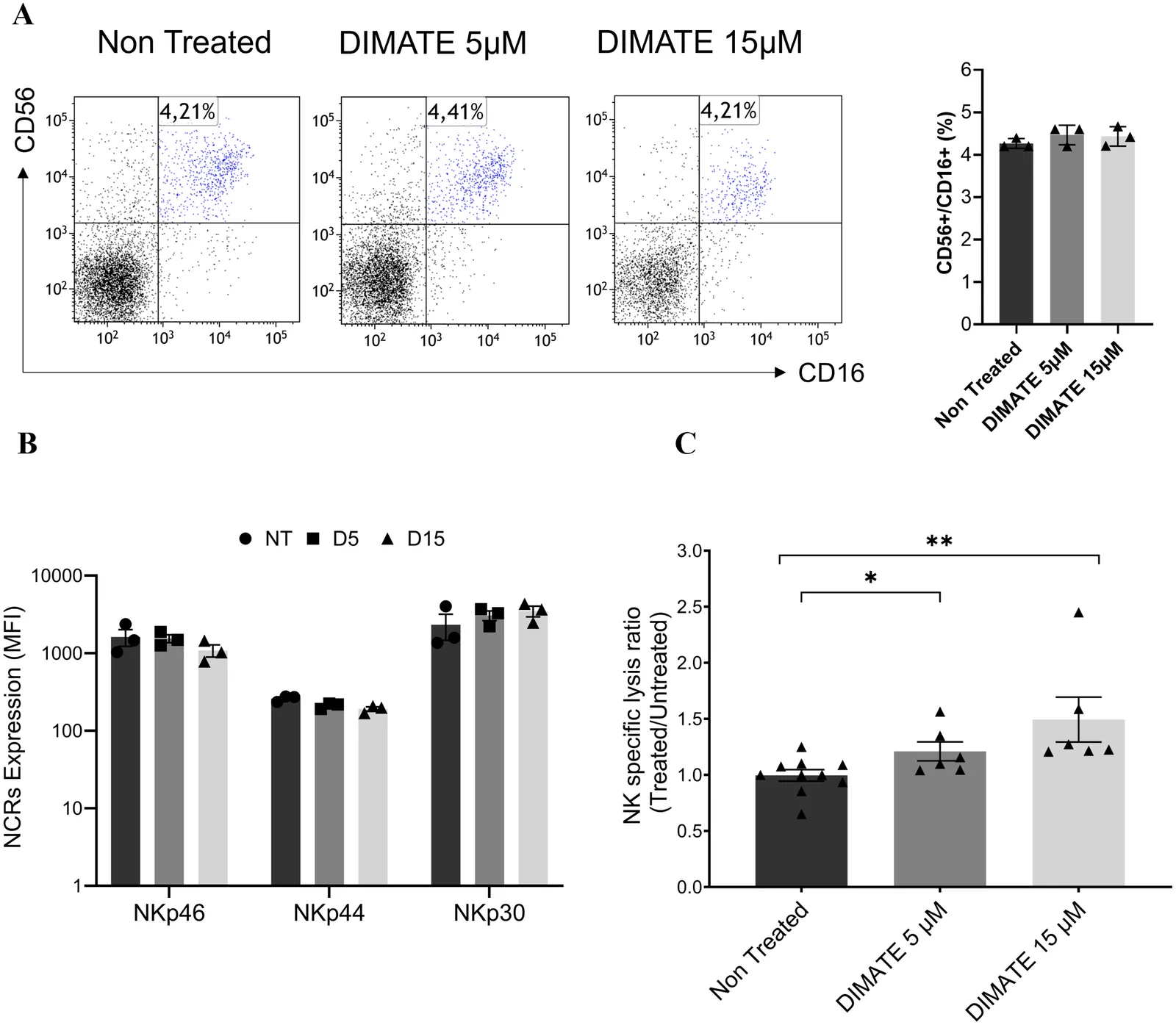
DIMATE effects on NK-cell proliferation, NK cytotoxicity. **(A)** NK-cell (CD56^+^/CD16^+^) frequency after overnight incubation with DIMATE at different concentrations: Non Treated (NT) 4.20% vs 5μM 4.60% (p = 0.417) and 15μM 4.42% (p = 0.526) (n = 3). Data are shown as mean ± SEM. Statistical analysis was performed using One-way ANOVA with Dunnett’s multiple comparisons versus NT. **(B)** Effect of DIMATE on the expression of natural cytotoxicity receptors (NCRs) on NK cells: NK cells were exposed to DIMATE (5 or 15 μM) and the expression of NKp46, NKp44 and NKp30 was assessed by flow cytometry. NKp46: NT 1620.8 ± 393.5; 5 μM 1542.9 ± 180.8; 15 μM 1084.0 ± 195.0. NKp44: NT 260.6 ± 12.9; 5 μM 211.1 ± 10.4; 15 μM 191.1 ± 11.8. NKp30: NT 2323.5 ± 853.6; 5 μM 3063.0 ± 437.4; 15 μM 3468.6 ± 545.7 (p = 0.31). Data represents geometric mean fluorescence intensity (gMFI) expressed as mean ± SEM (n = 3 independent donors). One-way ANOVA with Dunnett’s multiple comparisons versus NT. **(C)** NK cytotoxicity against MHC-I–negative K562 targets labeled with DiOC, measured by flow cytometry 18 h after co-incubation at an effector-to-target ratio of 200:1, following a 6h of DIMATE pretreatment (5 or 15 µM) or NT. Specific lysis ratios (treated/NT within the PI/DiOC double-positive gate) were: 5 µM = 1.21 ± 0.08 (p < 0.05) and 15 µM = 1.49 ± 0.20 (p < 0.01), compared with NT = 0.99 ± 0.08 (n = 6). Data are presented as mean ± SEM. Statistical analysis was performed using unpaired t-tests comparing each DIMATE concentration (5 and 15 µM) with NT.

In the NK-cell assay, DIMATE did not induce any major change in NK-cell frequency after overnight exposure, with comparable CD56+CD16+ populations in non treated (NT) and in exposed to 5 or 15 µM DIMATE (4.20%, 4.60% and 4.42% respectively; n = 3) (**Figure 3A**). NCR expression showed only modest, non-significant variations, including a decrease in NKp46 and NKp44 at 15 µM (1620.8 ± 393.5 vs 1084.0 ± 195.0 and 191.1 ± 11.8 vs 260.6 ± 12.9 respectively; n = 3) whereas NKp30 increased without reaching statistical significance at all doses (2323.56 ± 853.6 in NT vs 3468.6 ± 545.7 MFI at 15 μM; n = 3) (**Figure 3B**). In contrast, NK specific lysis (K562–DiOC assay) increased significantly, after 6 h DIMATE pretreatment across concentrations, with treated/NT ratio of 1.21 ± 0.08 at 5 µM and 1.49 ± 0.20 at 15 µM compared to NT 0.99 ± 0.08 (n = 6; p < 0.05) (**Figure 3C**). Overall, these exploratory data indicate that DIMATE enhances NK effector function despite stable NK proliferation and minimal changes in NCR expression.

### DIMATE engages a coordinated pro-inflammatory–ER-stress axis in AML cells

**Figure 4:**
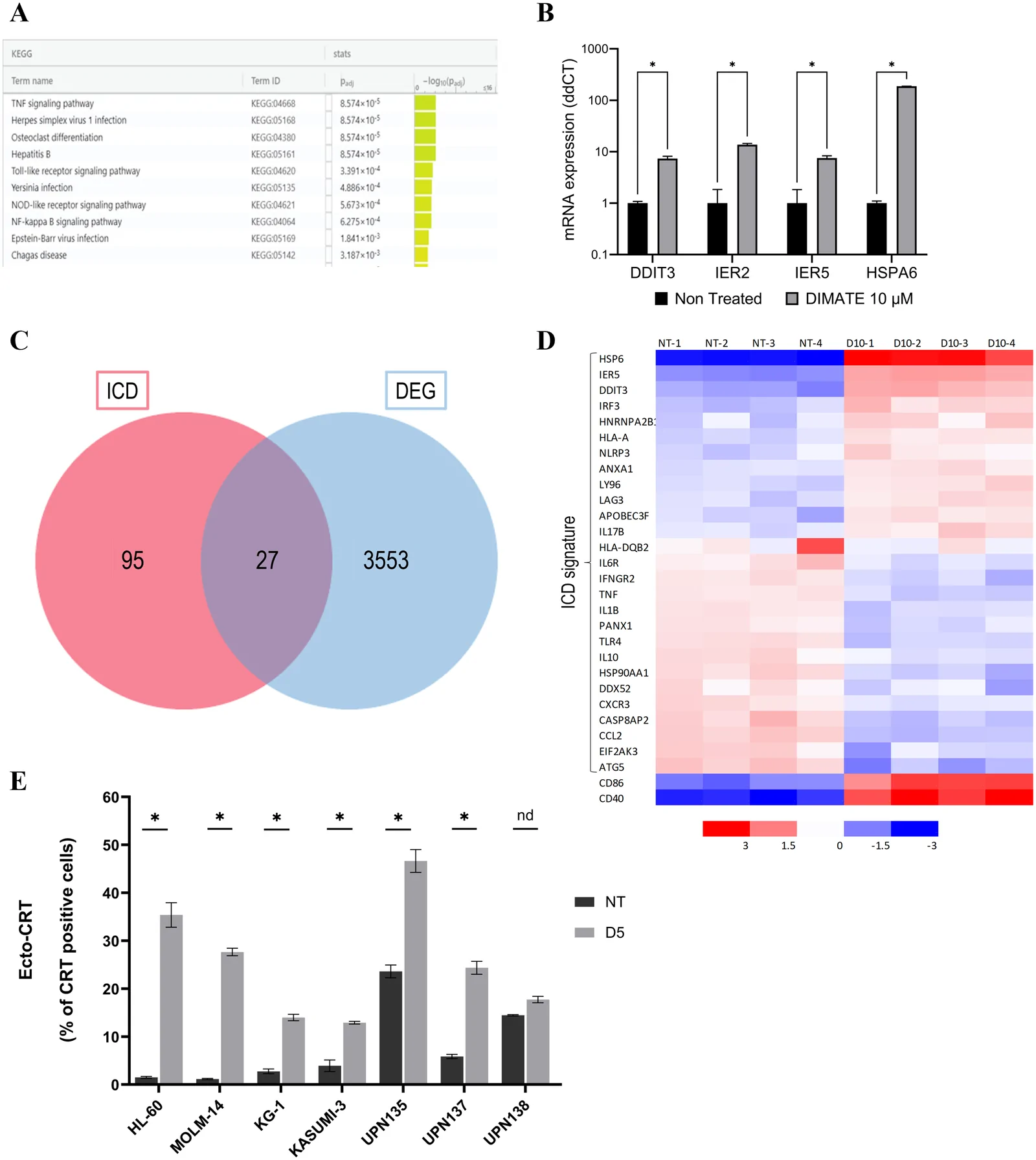
DIMATE induces ER-stress and an ICD-related transcriptional program in AML cells. **(A)** KEGG pathway enrichment of DEGs in HL-60 cells treated with 10 μM DIMATE for 6 h (Microarrays; fold-change ≥ 1.5, FDR ≤ 5%). Benjamini–Hochberg FDR control at 5%; g:Profiler KEGG enrichment with BH-adjusted (p) **(B)** RT-qPCR validation of ER-stress-related genes upregulated after DIMATE 10 μM during 6 h, including DDIT3 (CHOP) (P < 0.001), IER2 (P < 0.001), IER5 (P < 0.001), HSPA6 (P < 0.001) compared to Non Treated (NT) mRNA expression). Data are presented as mean ± SEM. Statistical analysis was performed using Paired, two-sided t-tests on ΔΔCt values vs NT with Benjamini–Hochberg correction across genes. (* = P < 0.001). **(C)** Venn diagram showing 27 genes intersecting between ICD-related gene sets obtained from Ping. et al., 2022 and DIMATE-induced DEGs **(D)** Heat map of ICD-associated genes (27 genes) in addition with CD86 and CD40 components in HL-60 treated with DIMATE 10 μM. Expression shown as row-scaled (z-score) log_2_ fold-change. **(E)** Surface calreticulin (ecto-CRT) exposure was quantified by flow cytometry under non-permeabilizing conditions. In HL-60, MOLM-14, KG-1 and KASUMI-3 AML cell lines after 24 h exposure to 5 µM DIMATE, treatment significantly increased ecto-CRT-positive cells in all models: HL-60 (1.50 ± 0.20% vs 35.39 ± 2.55%), MOLM-14 (1.15 ± 0.12% vs 27.67 ± 0.79%), KG-1 (2.75 ± 0.49% vs 13.98 ± 0.67%), and KASUMI-3 (3.92 ± 1.21% vs 12.92 ± 0.29%) (p < 0.0001; n = 3). In leukemic cells derived from AML patients, DIMATE treatment significantly increased ecto-CRT-positive cells: UPN135 (23,62 ± 1,34 vs 46,64 ± 2,37); UPN137 (5,87 ± 0,45 vs 24,36 ± 1,34) (p < 0.05; n = 3); except for UPN138 (14,46 ± 0,14 vs 17,73 ± 0,67) (p =0,34; n = 3). Data are shown as mean ± SEM. 2way ANOVA with Sidak’s multiple comparisons test was used for statistical analyses.

To probe tumor-intrinsic immune cues, HL-60 cells were treated with DIMATE (10 μM, 6 h) and profiled by Agilent microarray analysis, identifying 3554 DEGs at fold-change ≥ 1.5 and FDR ≤ 5%. Pathway enrichment highlighted TNF and Toll-like receptor (TLR) signaling—including the MyD88-independent (TRIF) branch (**Figure 4A**), together with increased expression of costimulatory molecules CD40 and CD86 (cf. **Figure 4D**), may indicate an innate inflammatory activation that can enhance antigen presentation and T-cell costimulation. Concomitantly, DIMATE significantly upregulated ER-stress markers (DDIT3/CHOP, ATF4, IER2, IER5, HSPA6), revealing a proteostatic stress response (**Figure 4B**).

### DIMATE aligns ER-stress induction with ICD-associated transcriptional and protein features

A Venn analysis identified 27 DEGs intersecting with an ICD signature (17) (**Figure 4C**). Across the enriched set, 52% were upregulated and 48% downregulated compared with controls (**Figure 4D**). The combined ER stress induction, ROS modulation, and ICD-associated transcriptional changes support a model in which DIMATE-induced cell death is compatible with ICD-associated remodeling. To corroborate ICD-associated danger signaling at the protein level, ecto-CRT exposure was assessed in four AML cell lines following DIMATE treatment. After 24 h exposure to 5 μM DIMATE, the proportion of ecto-CRT-positive cells significantly increased in HL-60 (1.50 ± 0.20% vs 35.39 ± 2.55%), MOLM-14 (1.15 ± 0.12% vs 27.67 ± 0.79%), KG-1 (2.75 ± 0.49% vs 13.98 ± 0.67%), and KASUMI-3 (3.92 ± 1.21% vs 12.92 ± 0.29%) cells (p < 0.05, n = 3). In addition to AML cell line, ecto-CRT is also increased in leukemic cells derived from AML’s patient UPN135 (23,62 ± 1,34 vs 46,64 ± 2,37); UPN137 (5,87 ± 0,45 vs 24,36 ± 1,34) (p < 0.05; n = 3); except for UPN138 (14,46 ± 0,14 vs 17,73 ± 0,67) (p =0,34; n = 3). The consistent induction of ecto-CRT observed in 86% AML models tested suggests that DIMATE triggers a conserved stress response leading to the exposure of a canonical ICD-associated “eat-me” signal (**Figure 4E**).

## Discussion

Therapy for hematologic malignancies has been transformed by immunotherapeutic approaches, particularly in B-cell malignancies and multiple myeloma In contrast, progress in AML has been considerably slower, except allogenic transplantation, owing to unique biological and therapeutic challenges that have limited the successful development of immune-based strategies (18–20). The persistence of MRD implies that eradicating the ultimate leukemic cells by cytotoxic treatment alone is challenging, if not unattainable; the host immune system must therefore contribute decisively to eliminate or at least restrain residual clones. Yet, most conventional regimens are highly immunosuppressive, motivating the development of agents that spare immune function, maintaining anti-infectious protection while retaining tumor-intrinsic cytotoxicity.

Building on the observation that DIMATE spares normal hematopoietic progenitors (8), we hypothesized that at therapeutic concentrations (~5 μM) DIMATE would not compromise immune responses and might, by virtue of ALDH inhibition, aldehyde accumulation and redox disruption, induce pro-immune signals in AML cells (9, 10, 13). Our data support this view, DIMATE induces no cytotoxicity in healthy PBMCs up to 20 µM. Phagocyte oxidative burst to opsonized bacteria was maintained. T cells (both CD4^+^ and CD8^+^) preserved short-term proliferation and early IL-2 production, with only transient, and reversible attenuation at the highest concentrations during prolonged exposure. NK cells retained baseline proliferation and showed enhanced NK specific lysis of MHC-I targets across doses (with significance at 5 and 15 μM), despite the absence of robust NCR modulation. Collectively, these findings suggest that DIMATE preserves selected functions of adaptive and innate immune effectors, even at concentrations exceeding the pharmacological goldilocks zone.

At first glance, this immune preservation may seem counterintuitive, given that DIMATE drives redox disequilibrium and cell death in tumor cells (13). However, redox biology is foundational to immune function (14, 21, 22). T-cells require physiological levels of ROS for TCR signaling, proliferation and memory formation, while antioxidant systems including the glutathione (GSH) and thioredoxin (TRX) pathways maintain redox homeostasis and prevent excessive oxidative damage (23). Central memory T-cells display metabolic programs associated with enhanced redox fitness and persistence in hostile microenvironments (24). Likewise, activated NK cells upregulate TRX-1 and peroxiredoxins, thereby increasing resistance to oxidative stress while preserving cytotoxic function (25, 26). Granulocytes are intrinsically adapted to high ROS production and deploy oxidative burst and NET formation as core effector mechanisms (27).

Beyond preservation, our tumor intrinsic analyses show that DIMATE induces some features associated with ICD in AML cells. NK cell mediated cytotoxicity seems to be upregulated when ALDH1A is inhibited in our work, this observation has been comforted in a recent study demonstrated that inhibition of ALDH1A1 enriched population with disulfiram, a pan-ALDH inhibitor, markedly enhanced NK-cell-mediated cytotoxicity through improved immunological synapse formation (28). RNA microarray analysis identified pro-inflammatory signaling, including TNF and Toll like receptor (TLR) pathways, with engagement of the MyD88 independent (TRIF) branch and up-regulation of co-stimulatory molecules (CD40, CD86), changes that may enhance antigen presentation and T-cell priming (29, 30). DIMATE also upregulated an ER-stress signature, consistent with ER stress–linked DAMP release, one of hallmark of ICD (13, 31). Mechanistically, oxidative and proteotoxic stress are established upstream triggers of ICD, in part through activation of ER stress responses and coordinated emission of DAMPs such as ecto-CRT exposure, ATP secretion and HMGB1 release, which can promote antigen-presenting cell activation and adaptive immune priming (31). In the present study, the detection of ecto-CRT after DIMATE exposure provides protein-level evidence consistent with the activation of an ICD-associated stress response in AML cells. However, because ATP secretion, HMGB1 release, antigen-presenting cell activation and T-cell priming were not directly assessed, these findings should be interpreted as supporting an ICD-compatible phenotype rather than demonstrating *bona fide* ICD. These findings have therapeutic implications. By preserving immune competence and inducing ICD-like features in AML cells, DIMATE could complement immune-engaging strategies, including antibody-based approaches and cellular therapies, and may be relevant for MRD control, where host immunity is critical (5, 6).

Clinically, the preservation of selected immune effector functions together with ICD-compatible tumor remodeling observed *in vitro* provides a rationale for incorporating exploratory immune monitoring into the ODYSSEY trial. Such monitoring could help relate DIMATE exposure to pharmacodynamic immune biomarkers, including ecto-CRT, ATP secretion, HMGB1, cytokines, and other inflammatory mediators. Infusion-related reactions (IRRs) have been reported to date (32). Although IRRs are clinically nonspecific and may arise from several non-mutually exclusive mechanisms, including hypersensitivity, cytokine release, treatment-related inflammation, or the underlying disease context, the immune-inflammatory profile induced by DIMATE *in vitro* provides a biologically plausible framework to explore their potential basis. In this context, one hypothesis is that, in a subset of patients, IRRs could reflect acute immune-inflammatory activation triggered by treatment exposure rather than representing purely nonspecific intolerance. However, this interpretation remains exploratory and should not be considered evidence of *bona fide* ICD, antitumor immune activation, or clinical efficacy *in vivo*. Dedicated prospective studies are therefore required to determine whether DIMATE-associated IRRs correlate with pharmacodynamic immune activation, including cytokine release, DAMP emission, TLR-related signaling, or other inflammatory biomarkers, and whether such signals have any relationship with clinical outcome.

More broadly, future studies should implement a comprehensive immune-monitoring strategy spanning ICD-associated biomarkers, antigen presentation, co-stimulation, checkpoint regulation, and effector function in primary AML samples, AML–dendritic cell/T-cell co-cultures, and *in vivo* models that recapitulate the tumor microenvironment. Functional co-culture assays using DIMATE-pretreated AML cells and untreated immune effectors will be required to determine whether ICD-compatible remodeling translates into enhanced immune-cell activation and leukemic cell killing. Within ODYSSEY, longitudinal evaluation of these markers at pharmacologically relevant exposures should help assess whether the immune-sparing and ICD-consistent signatures observed preclinically translate into measurable immune activation in patients. Ultimately, the ability of DIMATE to preserve immune function while inducing features compatible with ICD-like remodeling in AML cells may provide a rationale for bridging direct leukemic cytotoxicity with immune surveillance.

## Data Availability

The datasets presented in this study can be found in online repositories. The names of the repository/repositories and accession number(s) can be found in the article/**Supplementary Material**.

## Supplementary material

Supplementary Table 1 Clinical, cytogenetic, and molecular characteristics of primary AML patient samples.

Supplementary Figure 2 DIMATE promotes ecto-CRT exposure across AML cell lines and primary patient-derived AML samples. Representative flow cytometry analysis of ecto-CRT expression in AML cell lines (HL-60, MOLM-14, KG-1, and KASUMI-3) and primary AML samples (UPN135, UPN137, and UPN138) following treatment with DIMATE (5 µM), 24hours. Histograms show untreated cells (blue) and DIMATE-treated cells (red). Numbers indicate the percentage of ecto-CRT-positive cells.

Supplementary Table 3 Primer for qPCR.
